# Antifungal activity of alexidine dihydrochloride in a novel diabetic mouse model of dermatophytosis

**DOI:** 10.3389/fcimb.2022.958497

**Published:** 2022-09-02

**Authors:** Sunna Nabeela, Abhijit Date, Ashraf S. Ibrahim, Priya Uppuluri

**Affiliations:** ^1^ Division of Infectious Diseases, The Lundquist Institute for Biomedical Innovation at Harbor-University of California Los Angeles (UCLA) Medical Center, Torrance, CA, United States; ^2^ Department of Pharmacology and Toxicology, R. Ken (R. K.) Coit College of Pharmacy, University of Arizona, Tucson, AZ, United States; ^3^ Department of Ophthalmology and Vision Science, University of Arizona College of Medicine, Tucson, AZ, United States; ^4^ David Geffen School of Medicine at UCLA, Los Angeles, CA, United States

**Keywords:** dermatophytes, diabetes, alexidine dihydrochloride, ring worm, mouse model

## Abstract

Dermatophytosis is one of the most prevalent fungal infections and a major public health problem worldwide. Recent years have seen a change in the epidemiological patterns of infecting fungi, corresponding to an alarming rise in the prevalence of drug-recalcitrant dermatophyte infections. In patients with diabetes mellitus, dermatophytosis is more severe and recurrent. The potency of promising new antifungal drugs in the pipeline must be expanded to include dermatophytosis. To facilitate this effort, we established a clinically pertinent mouse model of dermatophyte infections, in which diabetic mice were infected with *Trichophyton mentagrophytes* on abraded skin. The diabetic mouse model was optimized as a simple and robust system for simulating dermatophytoses in diabetic patients. The outcome of infection was measured using clinical and mycological parameters. Infected mice with fungal lesions were treated with oral and topical formulations of terbinafine or topical administration of the FDA-approved and repurposed pan-antifungal drug alexidine dihydrochloride (AXD). In this model, AXD was found to be highly effective, with outcomes comparable to those of the standard of care drug terbinafine.

## Introduction

Dermatophytes are a group of fungi that cause superficial infections limited to the stratum corneum of the epidermis or to the hair and nails. *Trichophyton*, *Microsporum*, and *Epidermophyton* are the most common causes of dermatophytosis (de Hoog et al., 2017). While infections caused by these fungi are rarely life-threatening, they cause considerable morbidity, cosmetic embarrassment, and impose a significant financial burden. Dermatophytes are the foremost cause of cutaneous mycoses worldwide, prevalent in 20%–25% (or ~2 billion) of the global population (Hay et al., 2014; White et al., 2014). The United States alone records over 5 million outpatient visits due to dermatophytosis, with an annual burden of almost one billion dollars in associated direct medical costs (Brown et al., 2012).

While dermatomycoses can affect immunocompetent individuals, patients with diabetes mellitus are particularly susceptible to this infection (García-Humbría et al., 2005; Muller et al., 2005). Poor glycemic control and obesity are the top reasons for the high rates of infection in diabetic patients (Venturini et al., 2011; Celestrino et al., 2021). Of particular concern is the changing clinicoepidemiological scenario of dermatophyte infections, especially in tropical parts of Asia and Africa (Rippon, 1985; Coulibaly et al., 2017; Adebiyi and Gugnani, 2020). For example, this disease has been deemed an “epidemic” in tropical countries such as India, which has the second-largest population of diabetics (Rajagopalan et al., 2018). Dermatophytosis in India is attributed to rampant and irrational use of over-the-counter antibiotics and corticosteroid drug combinations (Verma et al, 2021; Verma and Madhu, 2017; Poojary et al., 2019; Das et al., 2020). In particular, recent years have seen a worsening in the disease severity with multiple, larger circumscribed inflammatory skin lesions harboring an overabundance of fungal loads similar to a biofilm-like etiology (Poojary et al., 2019). Furthermore, there has been a perceptible epidemiological shift in species from the previously predominant anthropophilic *T. rubrum* to *T. mentagrophytes* complex, a zoophilic fungus (Poojary et al., 2019; Adebiyi and Gugnani, 2020). Such a changing trend in etiology has paralleled the rate of increase in households harboring domestic pets, an important source of transmission (Segal and Elad, 2021). Making matters worse is the emergence of drug recalcitrance in these fungi, resulting in inevitable recurrences despite prolonged antifungal treatment (Martinez-Rossi et al., 2018; Khurana et al., 2019).

Topical and oral use of anti-dermatophytic drugs such as terbinafine and itraconazole have traditionally been the drugs of choice for ring worm infections (tinea corporis) caused by *Trichophyton* spp (Hainer, 2003; Singh et al., 2020). However, acquired resistance to these antifungal compounds is a rapidly emerging problem in developing countries (Monod et al., 2021). Indeed, there is a valid need for the discovery of novel drugs with enhanced effective and safe profiles.

Animal models of dermatophytosis have proven invaluable in evaluating the efficacy of antifungal molecules and for mechanistic understanding of fungal pathogenesis. Although guinea pigs have been the most commonly used animals in studies of dermatophytosis due to their likeness to human skin (Saunte et al., 2008; Shimamura et al., 2012), their use suffers from several shortcomings, including the lack of knockout animals. To circumvent this limitation, studies have exploited the mouse model for experimental dermatophytosis to understand disease pathology and host response (Hay et al., 1988; Nakamura et al., 2012; Baltazar Lde et al., 2014). We and others have extensively reported on the advantages of using mice in fungal infections, including their affordability, relative ease of use, and amenability to induce various disease conditions such as immunosuppression or diabetes (Capilla et al., 2007; Sano et al., 2014; Uppuluri et al., 2018; Gebremariam et al., 2019; Gebremariam et al., 2021). Thus, in establishing a new animal model for dermatophytosis, we considered three main factors: diabetic predisposition for clinical relevance; most prevalent zoonotic dermatophyte; and a model simple and affordable enough to use in research laboratories equipped for small rodents. Here, we optimized a diabetic mouse model of dermatophytosis, yielding a clinical picture akin to that observed in humans (Gebremariam et al., 2021). We further harnessed this simple, robust, and reproducible model to test the efficacy of a standard of care antifungal agent, terbinafine, and a new broad-spectrum antifungal molecule, alexidine dihydrochloride (AXD), recently discovered by our group (Mamouei et al., 2018). Our results show that the efficacy of AXD was analogous to that of terbinafine, resulting in complete clinical and mycological cure compared to infected untreated controls.

## Results and discussion

### Assessment of progression of infection

**Figure 1:**
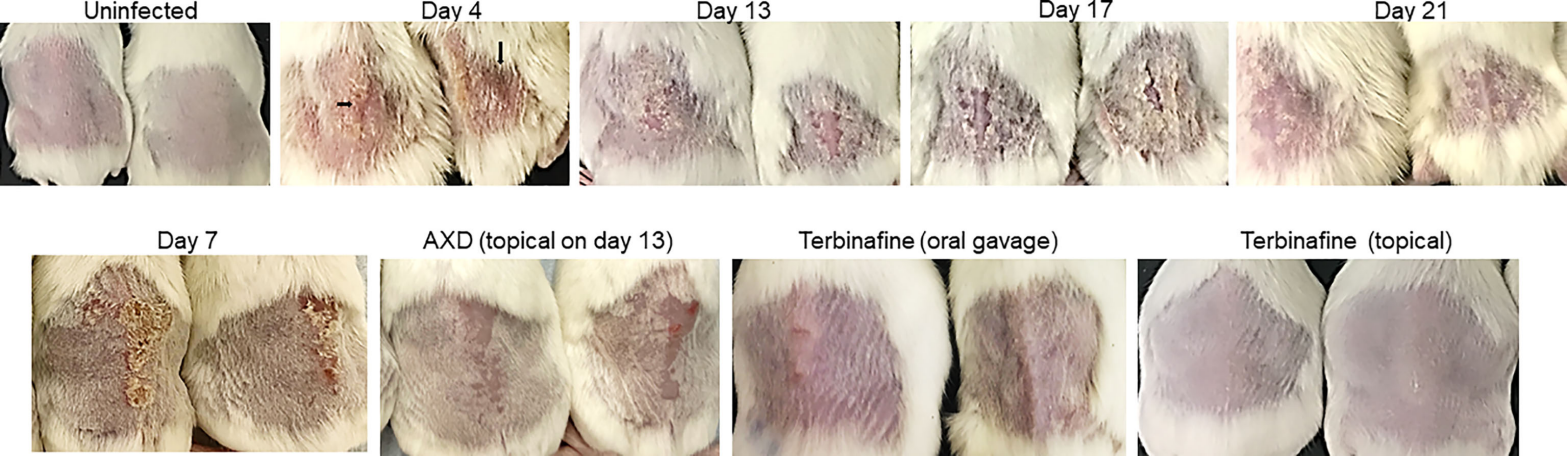
Clinical picture of dermatophytosis by *T. mentagrophytes* on the skin of mice and efficacy of antifungal drugs: Skin of the back of diabetic mice were infected with 1 × 10^7^ conidia and the clinical picture of infection was monitored over time. Arrows indicate redness and edema. Infected skin at day 7 was also treated topically with AXD (20 µg) or terbinafine (1% topical, or oral gavage 75 mg/kg). Observe the complete clearance of hyperkeratosis and redness post treatment with both drugs.

**Figure 2:**
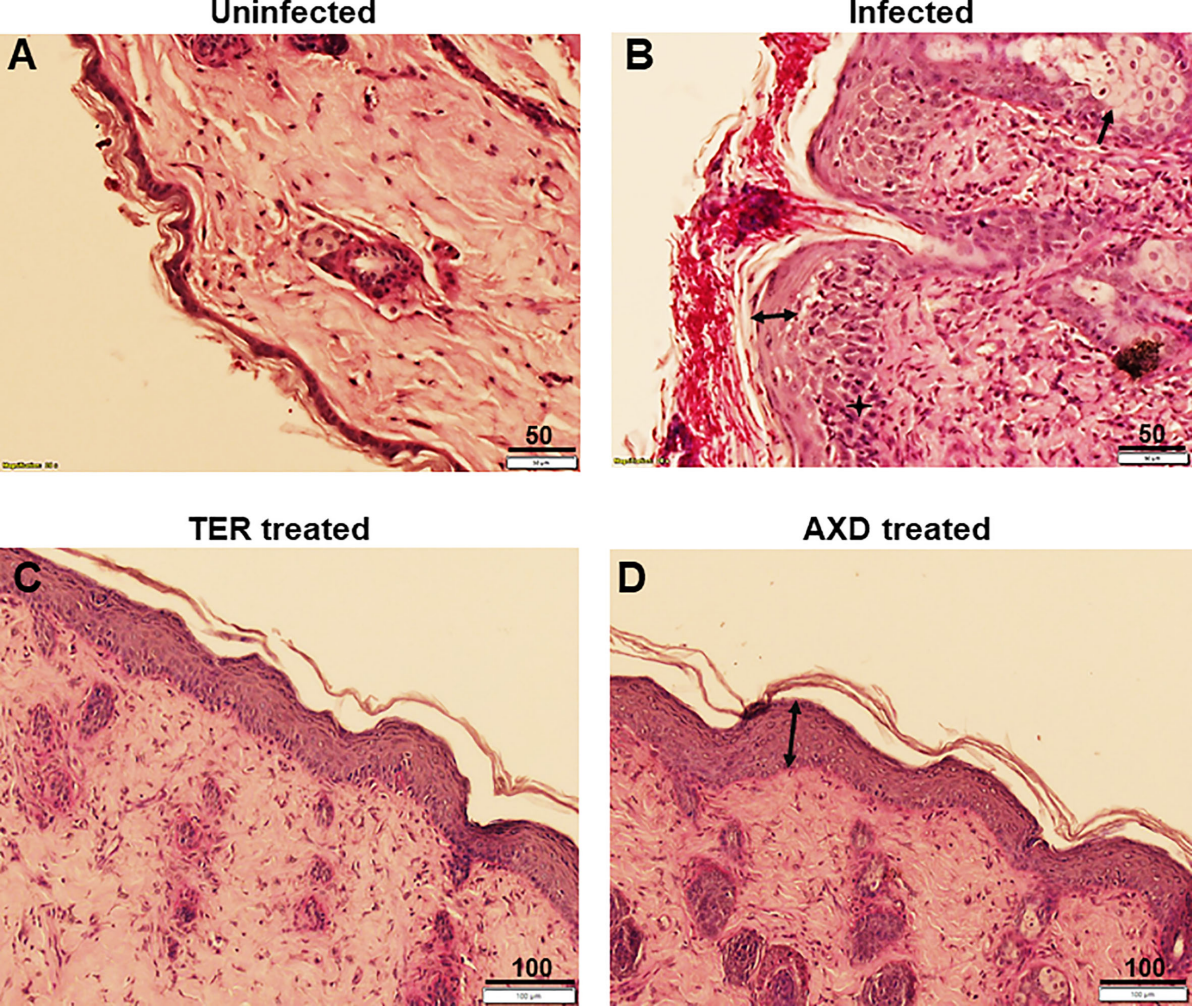
Histopathological analysis of skin: Skin biopsies obtained from uninfected, infected or drug treated mice skin were fixed, sectioned and stained with H&E plus PAS stain. **(A)** shows structure of intact skin from uninfected control, **(B)** exhibits infection, and presence of extensive hyphae on the epidermal layer. Infection causes acanthosis (two headed arrow) and spongeosis (arrow), **(C, D)** show complete clearance of hyphae from the skin, and regeneration of the stratum corneum (although acanthosis is still observed). Scale bars as indicated in µm.

The pathophysiology of diabetic mice infected with *T. mentagrophytes* (ATCC26323) was monitored over time. The overall success rate of infection in our studies was 100%, based on clinical and mycological outcomes (**Table 1**; 17 of 20 mice were successfully infected). Mice that resisted infection turned out to be those that did not develop diabetes (<250 mg/dl urine glucose). The earliest signs of infection appeared between days 3 and 4, post infection when the skin of mice was visibly red and erythematous (**Figure 1**). The lesions gradually became worse by 7 to 13 days, post-infection, and exhibited plaque-like erythema, edema, and hyperkeratosis. Fungal infection was confirmed by skin scraping followed by culturing, which revealed *T. mentagrophytes* in all animals on days 4, 7, and 13 (**Table 1**). Interestingly, while the hyperkeratotic lesions persisted up to day 17, the culture positivity rates were reduced to 60%, indicating that the infection was starting to resolve. Accordingly, by day 21, shedding of skin crusts led to a significant visible reduction in hyperkeratosis (p <0.01 versus other time points; **Figure 1**) and a further reduction in fungal growth to 40% (**Table 1**). The histopathology of skin sections from day 13 revealed a normal stratum corneum, while that of the infected skin displayed signs of inflammation characterized by acanthosis (thickness/hyperplasia of the epidermis), spongiosis (edema in the epidermis), and moderate cellular infiltration (**Figures 2A, B**). **Supplementary Figure S1** shows the fungal filaments penetrating the hair follicles (arrow) and immune infiltration around the area of infection (star; also enlarged). Additionally, only the infected areas show acanthosis (two-sided arrows), while the uninfected loci of skin taper back to normal. The presence of marked dermal edema, acanthosis, and cellular infiltrates predominantly composed of mononuclear cells has been frequently elucidated in dermatophytosis (Hay et al., 1983; Nantel et al., 2002; Saunte et al., 2008).

**Table 1:** Clinical and mycological assessments of infection during model establishment and after antifungal drug treatment.

Infection progression in diabetic mice				
Days	# mice	Erythema	Hyp.ker	%Culture +
4	20	2.7	0.3	88
7	20	1.85	2.4	100
13	20	0.45	2.8	100
17	20	0.15	2.6	60
21	20	0	1.8	40
**Efficacy of AXD and TER**				
**Drug**	**% reduced erythema**	**p value**	**% mycological efficacy**	**p value**
Vehicle (gel)	0%	x	0%	1
AXD (T)	83.33%	0.0003	100%	<0.0001
TER (T)	91.66%	0.0002	100%	<0.0001
TER (O)	87.5%	0.0002	100%	<0.0001

There was statistical significance (p <0.01) between all the time points in terms of erythema scores. Again, p <0.01 between day 4 and the rest of the time points (days 7 to 21) for hyperkeratosis. AXD, Alexidine dihydrochloride; TER, terbinafine; T, topical; O, oral gavage; %mycological efficacy, culture negativity.

Disease progression in the diabetic mouse model paralleled that of previous studies established in the immunocompetent guinea pig model. However, the presence of diabetes as a physiologically relevant-predisposing factor induced far more severe lesions, peaking at days 7–10 versus days 10–15 in other published models (Saunte et al., 2008; Fontenelle et al., 2014). In fact, such erythematous and hyperkeratotic manifestations are frequently witnessed in diabetic patients or those receiving immunosuppressive corticosteroid treatment (Fraga-Silva et al., 2015; Poojary et al., 2019). In a parallel control arm, when immunocompetent mice were infected with the dermatophyte, they displayed significantly mild erythema and dryness (**Supplementary Figure S2A**), with only 10% of the mice culture positive and clearance of fungi by day 17 (data not shown).

Hyperglycemic mice have previously been used to investigate the host response to dermatophytosis (Venturini et al., 2011; Fraga-Silva et al., 2015; Almeida et al., 2017). However, these studies did not elaborate on the clinical picture of infection or the amenability of the model to evaluate drug efficacy. Besides, all these studies induced type I diabetes using the drug alloxan, which is toxic to the pancreas and can lead to severe debilitation and mortality in mice. Our studies used streptozotocin, which has a high inductive capacity, less toxicity, and more specificity for pancreatic beta cells than alloxan (Lenzen, 2008).

### Testing efficacy of antifungal compounds in the diabetic mouse model of dermatophytosis

We also used the developed diabetic mouse model for pre-clinical evaluation of alexidine dihydrochloride (AXD), an FDA-approved repurposed molecule, recently identified by us to have a broad-spectrum activity against pathogenic fungi (Mamouei et al., 2018). In this study, the MIC_80_ of AXD *in vitro* was determined to be 0.32 µg/ml against *T. mentagrophytes* and 0.64 µg/ml versus *T. rubrum*, two of the most commonly isolated dermatophyte species from tinea infections (**Supplementary Figure S2B**). This dose of AXD has previously been shown by our laboratory to be effective against other human fungal pathogens, including *Candida albicans* and *Cryptococcus neoformans* (Mamouei et al., 2018). The MIC of terbinafine against *T. mentagrophytes* was 0.008 µg/ml, a concentration previously shown by us and others to be effective against the fungus.

Since the peak of infection in this model was between days 7 and 12, mice were treated with antifungal drugs for 6 days starting on day 7 of the infection. To facilitate the delivery of AXD onto the skin of mice, we followed a strategy previously reported by us, where AXD was incorporated into an *in situ* gelling formulation composed of 20% w/v P407 and 1% w/v Poloxamer 188. We and others have demonstrated that thermosensitive P407 hydrogels can be effectively used for the intra-oral and intra-vaginal application of nanoparticles without affecting their inherent properties and release (Date et al., 2012; Date et al., 2015; Chen et al., 2021; Liu et al., 2021). Furthermore, studies have shown that Poloxamer 407 thermosensitive hydrogel can also potentiate delivery and sustained release of antimicrobials for improved efficacy against microbial biofilms (Bernegossi et al., 2020; Lp et al., 2020; Liu et al., 2021). Hence, P407 thermosensitive gel containing AXD was deemed suitable for topical use. Terbinafine (TER) was used as a positive control since this drug is the standard of care drug for the treatment of dermatophytosis (although resistance to terbinafine is emerging) (Babu et al., 2017; Nenoff et al., 2020).

Oral and topical applications of terbinafine as well as topical applications of AXD yielded similar mycological and clinical clearance of infection. On day 7 post-treatment (i.e., day 13 post-infection), lesions were completely healed with a striking reduction in infection post topical treatment with the two drugs (**Figure 1**). Besides the visual inspection, the efficacy of the two drugs was also confirmed by monitoring clinical and mycological efficacy. Whereas the infected and untreated mice demonstrated skin regions and culture positivity, mice treated with AXD and TER displayed significantly reduced erythema (>83%–91% efficacy; p <0.0001; **Table 1**) and a complete absence of fungal growth on culture, indicating 100% mycological efficacy post-treatment (**Table 1**). Histopathological analysis on day 13 of infected skin treated with topical terbinafine or AXD confirmed complete clearance of fungi from all mice (**Figures 2C, D** **;** **Supplementary Figure S3**). Even after scrutinizing multiple sections tested from skin specimens of several different mice, the worst-case scenario found was a single focus of very few residual hyphal cells (**Supplementary Figure S2C**). Considering that these mice were culture negative, it is likely possible that these filaments are inviable. Alternatively, it could also be that these isolated hyphal strands escaped treatment and therefore could be considered as “persister cells” that could reinitiate growth and cause a relapse of infection or drug recalcitrance. Despite treatment with the two drugs, hyperplasia and edema of the epidermis were not reversed (**Figure 2D**, double-sided arrow). Such inflammation parameters have also been observed previously in the guinea pig model of dermatophytosis. Future studies could be undertaken to investigate if, or when the epidermis reverts to its normal architecture, a reappearance of infection once treatment is stopped. Such is often the case in human infections, where recurrence occurs weeks or even months after the course of treatment is completed.

Our results for terbinafine efficacy are in agreement with the findings of Ghannoum et al., who demonstrated that both topical and oral preparations of terbinafine are 90%–100% potent against *T. mentagrophytes* in a guinea pig model (Ghannoum et al., 2004; Saunte et al., 2008). Terbinafine has the propensity to efficiently bind keratinocytes, rapidly penetrate the stratum corneum, and persist in the skin at concentrations multifold higher than its MIC *in vitro* (Shear et al., 1991; Faergemann et al., 1993). This is the first study to evaluate the efficacy of AXD in an animal model of dermatophytosis. AXD, a member of the bisbiguanide class of antiseptics, has been noted as an anticancer drug lead because of its apoptotic activity *in vitro* and *in vivo* (Yip et al., 2006). Furthermore, this compound has been tested as an antiplaque agent and mouthwash with the potential to be used in endodontic treatment to eliminate biofilms (Lobene and Soparkar, 1973; Spolsky and Forsythe, 1977; Kim et al., 2013; Ruiz-Linares et al., 2017). Interestingly, in all these applications, AXD is potent at concentrations manifold higher than the MIC80 demonstrated in this study. This accentuates the potential of AXD as a stellar anti-dermatophytic drug. Indeed, our previous report highlighted the potential of AXD as a broad-spectrum antifungal drug with activities against biofilms and azole-resistant fungi, while exhibiting low mammalian-cell toxicity (Mamouei et al., 2018). As a next step in this series of investigations, it will be important to examine the pharmacokinetics and biodistribution of AXD on the skin.

In conclusion, we have presented a simple, physiologically relevant animal model that mimics infections in humans with *T. mentagrophytes* and harnessed this model to unravel the stellar activity of a novel molecule, AXD, against dermatophytosis. This diabetic mouse model can be applied for efficacy testing of new antifungals against dermatophytes, evaluation of diagnostic candidates, or to study the less understood host response to dermatophytoses in the background of hyperglycemia.

## Methods

### Fungal strain and growth conditions

The dermatophyte strain *Trichophyton mentagrophytes* ATCC 26323 was used throughout this study. This is a virulent clinical isolate from an aggressive ringworm infection isolated from a patient in Vietnam (Hashimoto et al., 1972; Hashimoto and Blumenthal, 1977; Suh et al., 2018). Dermatophytes were subcultured from the primary Sabouraud agar plate (containing 0.4 g/L cycloheximide and 0.5 g/L chloramphenicol; SD+ agar) to oat meal agar medium to induce conidiation. Plates were incubated at 35°C for 7 days or longer until colonies developed abundant spores. The conidial spores were then carefully collected by gently flushing 5 ml of phosphate buffer saline (pH = 7.4) on top of the colonies and aspirating the suspension into a sterile collection tube. Fungal spores were enumerated using a hemocytometer.

### Antifungal agents

Terbinafine for oral gavage treatment was obtained from Novartis (Summit, NJ) and Terbinafine 1% cream (Lamisil™) was obtained commercially. Alexidine dihydrochloride powder was obtained from Sigma (St. Louis, MO). Both drug powders were dissolved at a concentration of 1 mg/ml in 10% DMSO. For the preparation of alexidine thermosensitive gel, 10 mg of AXD was dissolved in 10 ml of water using a vortex mixer and ultrasonic bath. Poloxamer 407 (2 g) and Poloxamer 188 (100 mg) were then dispersed into AXD solution with the help of a vortex mixer and the dispersion was stored overnight in the refrigerator to dissolve Poloxamer 407 and Poloxamer 188, leading to an *in situ* gelling formulation containing AXD. The P407 *in situ* gelling formulation containing AXD was stored in the refrigerator until further use.

### Animal model

All animal-related study procedures were compliant with the Animal Welfare Act, the Guide for the Care and Use of Laboratory Animals, and the Office of Laboratory Animal Welfare and were conducted under an IACUC approved protocol 31789-01 by The Lundquist Institute at Harbor-UCLA Medical Center. Male ICR mice (20 to 23 g) were rendered diabetic with a single intraperitoneal injection of 210 mg of streptozotocin/kg of body weight in 0.2 ml of citrate buffer 10 days prior to the fungal challenge, as we have previously described (Ibrahim et al., 2003). This dose of streptozotocin causes diabetes in 80 to 90% of the injected mice. Glycosuria and ketonuria were determined with keto-Diastix reagent strips (Bayer, Elkhart, Ind.) 7 days after streptozotocin treatment. Consistent with the establishment of DKA, diabetic mice had a decrease in blood pH from 7.8 (normal for mice) to 7.3–7.2, associated with increased levels of urinary glucose (moderate increase of 250 mg/dl to a high level of >1,000 mg/dl) and urinary ketone bodies (moderate levels of 2 to 4 mg/dl to a high concentration of ≥5 mg/dl) as determined by Keto-Diastix strip testing. Mice were anesthetized by i.p. injection of 0.2 ml of a mixture of ketamine at 82.5 mg/kg (Phoenix, St. Joseph, MO) and xylazine at 6 mg/kg (Lloyd Laboratories, Shenandoah, IA). The sedated mice were kept on heat pads (Fisher Scientific) which were prewarmed to 37°C. The backs of the mice were shaved using an electric shaver. As reported previously (Ghannoum et al., 2004; Saunte et al., 2008), a 3 × 3 cm area on the shaved skin was scraped gently with sandpaper to disturb the epidermidis and infected with 5 × 10^7^ cells/ml of *T. mentagrophytes* conidia. For infection, 50 µl of PBS containing the spores was applied and rubbed on the skin of mice using a pipette tip until the application dried on the skin. Uninfected control mice skin was applied with PBS. For drug treatment, 7-day infected mice were treated with oral terbinafine (75 mg/kg), and the entire surface of the skin was applied with 1% topical terbinafine or AXD topical thermosensitive gel (20 µg). Treatment was continued once daily for 6 days.

### Evaluation of the outcomes of infection

For clinical assessments, the skin of 20 mice was visually monitored daily for erythema (E), crusting or hyperkeratosis (H), at various time points post-infection (0 to 17 days). Due to hyperglycemia, the infection was severe and reproducible. The clinical parameters for erythema were scored blindly as the following: none 0, mild or spotty 1, well defined 2, or inflamed 3. Additionally, dryness and crusting were scored similarly, with mild dryness at 1 and hyperkeratosis at 3. Thus, the clinical score had a range from 0 (no infection) to 3 (worst outcome). These scores were used to compare the efficacy of the antifungal drugs. Percent efficacy was calculated as 100 − (T/C × 100) where T = the total score of the treatment group and C = the total score of the untreated control (infected) group. Total score = average clinical score from animals in the same group. One-way ANOVA or Student’s t-test was used to analyze data using Graphpad prism software (p <0.05 was considered significant).

Mycological assessments were performed in a repeat set of experiments with 20 infected mice. The skin was scraped from parts of the infectious lesion using a sterile scalpel, and the skin dust, as well as 10 uprooted hairs, was collected in an empty Petri dish. Specimens were used for culture on SD+ plates, incubated at 35°C for 3 days, and fungal growth was monitored. The presence of even one colony of fungus was considered culture-positive.

### Histopathology

Skin samples were obtained from three animals per group on day 13 of the study. Skin (~1 cm^2^) was excised using sterile scissors from sacrificed animals. Skin samples were fixed in zinc-buffered formalin, embedded in paraffin, sectioned, and stained with H&E and PAS for visualization of the epidermis and fungal morphology, respectively.

### *In vitro* MIC testing

An antifungal susceptibility assay was performed by following the Clinical and Laboratory Standards Institute (CLSI) guidelines, document M38-A2 for filamentous fungi (Wayne, 2008). Conidial spores were isolated as described above and used at a final density of 1 to 3 × 10^3^ cells/ml for testing. The concentration range of terbinafine evaluated was 0.001–0.5 μg/ml; and that of AXD was 0.02–20 μg/ml. The minimal inhibitory concentrations (MICs) were defined as the lowest concentrations that led to complete inhibition of observable growth of *T. rubrum* and *T. mentagrophytes* after 4 days.

## Data availability statement

The raw data supporting the conclusions of this article will be made available by the authors, without undue reservation.

## Ethics statement

The animal study was reviewed and approved by The Lundquist Institute at Harbor-UCLA Medical Center.

## Author contributions

SN performed all studies. AD provided the thermogel formylations of AXD. AI contributed to the concept, troubleshooting, and editing the manuscript. PU was responsible for the concept, design, experimentation, writing, and editing the manuscript. All authors listed have made a substantial, direct, and intellectual contribution to the work and approved it for publication.

## Funding

We would like to thank the following agencies for their financial support to carry out this project: the NIH NIAID R01AI141794 awarded to PU, the NIAID 1R01AI141202-01 awarded to AI, and the NIH NIGMS P20GM103466 awarded to AD.

## Conflict of interest

The authors declare that the research was conducted in the absence of any commercial or financial relationships that could be construed as a potential conflict of interest.

## Publisher’s note

All claims expressed in this article are solely those of the authors and do not necessarily represent those of their affiliated organizations, or those of the publisher, the editors and the reviewers. Any product that may be evaluated in this article, or claim that may be made by its manufacturer, is not guaranteed or endorsed by the publisher.

## Supplementary material

Supplementary Figure S1 Hyphal invasion of the epidermis: Extensive hyphal invasion of the stratum corneum (thick arrow) on day 13 of *T. mentagrophytes*, which causes acanthosis (two headed arrow) and infiltration of inflammatory cells (star) at the site of infection (see inset for a zoomed in view).

Supplementary Figure S2 MIC of AXD *in vitro*: **(A)** development of mild infection in immunocompetent mice. **(B)** Picture of MIC of AXD against T. mentagrophytes (TM) and *T. rubrum* (TR). **(C)** A single locus of residual hyphae post drug treatment shows an almost complete clearance of infection.

Supplementary Figure S3 Skin histology images post drug treatment: Zoomed out (Magnification 2x) images of surface area of skin to visualize clearance of infection in TER and AXD treated mice. Scale bars = 500 µm.
