# Supplementary figures and images for: Antifungal activity of alexidine dihydrochloride in a novel diabetic mouse model of dermatophytosis
Source: Front Cell Infect Microbiol. 2022 Sep 2;12:958497. doi: 10.3389/fcimb.2022.958497 (PMC9478942; doi:10.3389/fcimb.2022.958497)

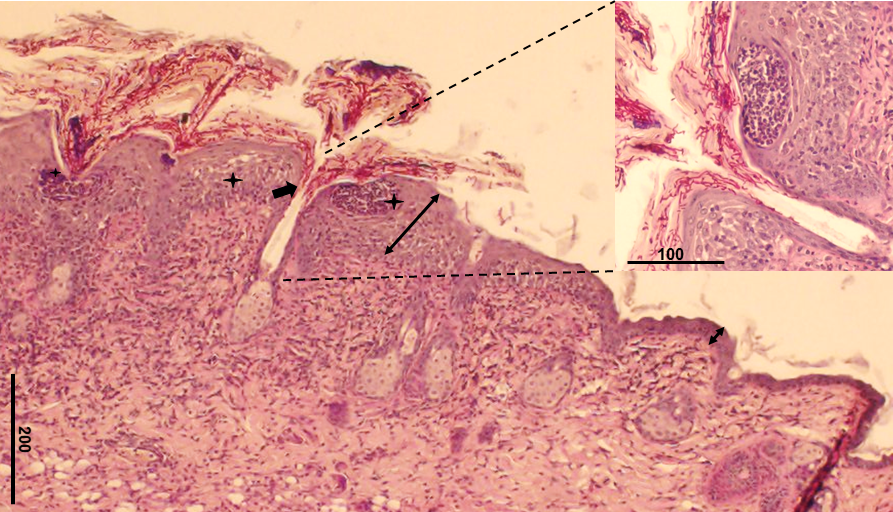

Supplement: Supplementary Figure S1 — Hyphal invasion of the epidermis: Extensive hyphal invasion of the stratum corneum (thick arrow) on day 13 of T. mentagrophytes, which causes acanthosis (two headed arrow) and infiltration of inflammatory cells (star) at the site of infection (see inset for a zoomed in view). [file DataSheet_1.zip › S1.TIF]

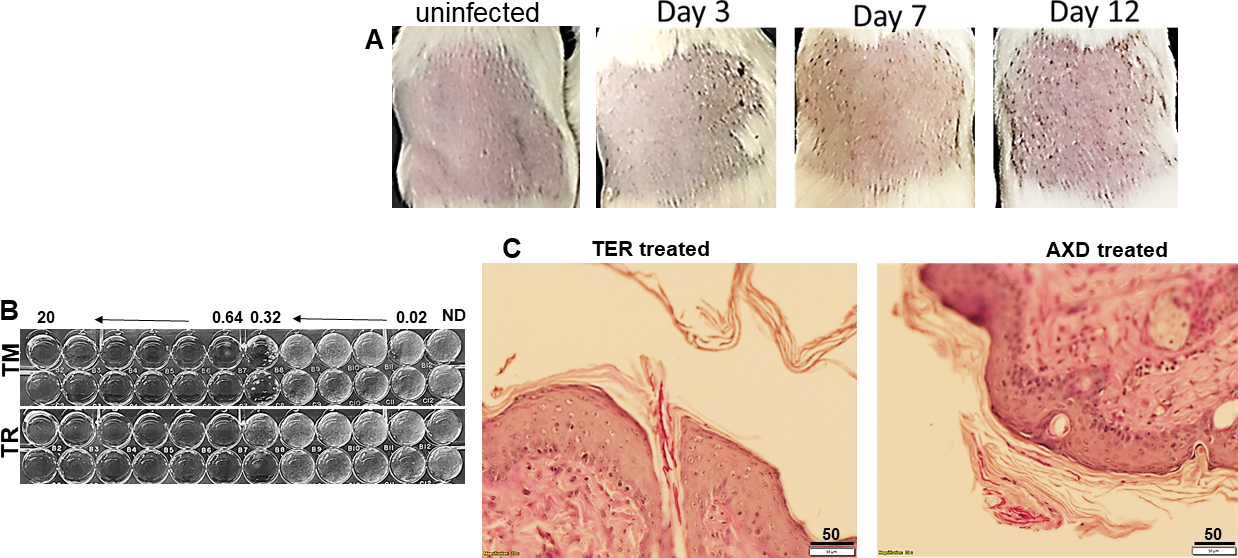

Supplement: Supplementary Figure S1 — Hyphal invasion of the epidermis: Extensive hyphal invasion of the stratum corneum (thick arrow) on day 13 of T. mentagrophytes, which causes acanthosis (two headed arrow) and infiltration of inflammatory cells (star) at the site of infection (see inset for a zoomed in view). [file DataSheet_1.zip › S2.tif]

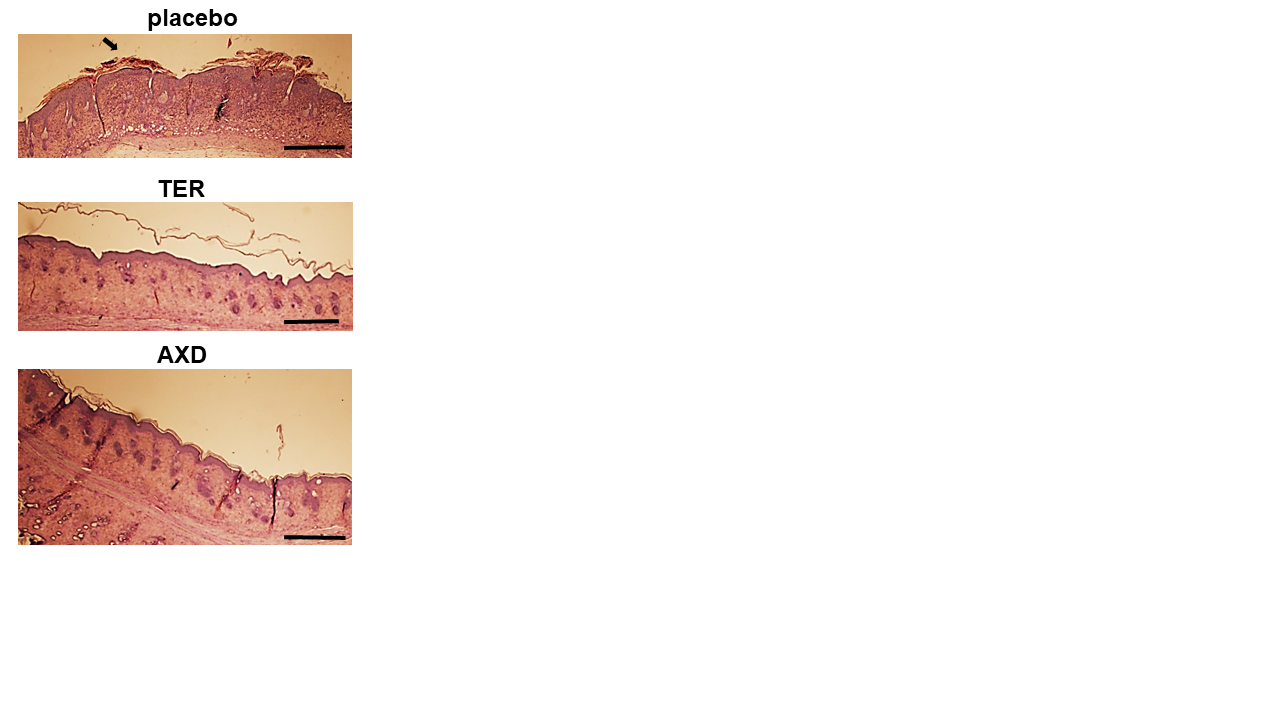

Supplement: Supplementary Figure S1 — Hyphal invasion of the epidermis: Extensive hyphal invasion of the stratum corneum (thick arrow) on day 13 of T. mentagrophytes, which causes acanthosis (two headed arrow) and infiltration of inflammatory cells (star) at the site of infection (see inset for a zoomed in view). [file DataSheet_1.zip › S3.tif]
